# Microplastics and bisphenol A in the edible mussel *Perna perna* from southeastern Brazil: occurrence and dietary exposure assessment

**DOI:** 10.1007/s10661-026-15982-y

**Published:** 2026-10-23

**Authors:** Dandara Silva Cabral, Gabriel Possé Santos de Abreu, Mércia Barcellos da Costa, Stanley Lohan Nichel Alves, Gustavo Zambon Dalbó, Bruno Quirino Araújo, Enrique Ronald Yapuchura Ocaris, Antonio Augusto Lopes Marins, Fabian Sá

**Affiliations:** 1 Environmental Geochemistry Laboratory (LabGam), Federal University of Espírito Santo (UFES), Vitória, Espírito Santo Brazil; 2 Coastal Biology and Microplastic Analysis Laboratory (LaBCAM), Federal University of Espírito Santo (UFES), Vitória, Espírito Santo Brazil; 3 Chromatography Laboratory, Federal University of Espírito Santo (UFES), Vitória, Espírito Santo Brazil; 4 Escuela Profesional de Ingeniería de Minas, Universidad Católica de Santa María de Arequipa, Arequipa, Peru; 5 Multiusual Laboratory of Instrumentation (LabMIinst-LabPetro), Chemistry Department, Federal University of Espírito Santo (UFES), Vitória, Espírito Santo Brazil

**Keywords:** Microplastics, Bisphenol A, Mussel, Seafood safety, Dietary exposure, Risk assessment, Shellfish

## Abstract

Edible bivalves are an important route of human exposure to environmental contaminants because they are consumed whole and accumulate particulate and chemical hazards from coastal waters. This study assessed microplastics (MPs) and bisphenol A (BPA) in the brown mussel *Perna perna* from Espírito Santo Bay, southeastern Brazil, and estimated dietary exposure under average- and high-consumption scenarios. Sixty mussels were analyzed: 30 for MPs and 30 for BPA, of which 18 yielded valid quantitative BPA results. MPs were detected in all analyzed mussels, with a mean abundance of 1.44 ± 1.19 MPs g⁻^1^ wet weight (ww). Fibers, mainly blue and black, were the predominant morphotype of MPs. BPA concentrations ranged from 4.80 to 7.25 μg kg⁻^1^ ww. Under average consumption, BPA intake was below the current European Food Safety Authority tolerable daily intake (EFSA TDI), with an estimated daily intake of 0. 0118 ng kg⁻^1^ bw day⁻^1^ and a hazard quotient (HQ) of 0.06. Under high consumption, intake increased to 2,46 ng kg⁻^1^ bw day⁻^1^, corresponding to an HQ of 12.30 and exceeding the EFSA TDI of 0.2 ng kg⁻^1^ bw day⁻^1^. Together, the detection of MPs and BPA in separate subsets of the sampled mussel population provides complementary evidence of particulate and chemical plastic-related contamination in edible shellfish, without implying a direct association between the two contaminants. These findings indicate that edible mussels may be a relevant dietary exposure route to plastic-related contaminants, especially for the frequent consumers, and support the inclusion of emerging contaminants in food safety surveillance of coastal shellfish.

## Introduction

Edible bivalves are increasingly recognized as relevant food matrices for the assessment of human exposure to environmental contaminants because they are consumed as whole soft tissues and can accumulate both particulate and chemical hazards from coastal waters (Khanjani et al., 2023; Van Cauwenberghe & Janssen, 2014; Xu et al., 2024). Among these contaminants, microplastics (MPs) have received growing attention due to their widespread occurrence in aquatic environments and their presence in seafood intended for human consumption. Their persistence, small size, and bioavailability make them particularly relevant for food safety studies involving filter-feeding organisms (Alimba & Faggio, 2019; Parashar & Hait, 2023a, b). These contaminants are increasingly relevant to food safety because they can accumulate in edible aquatic tissues and contribute to human dietary exposure (Parashar & Hait, 2023a, b).

Bivalve mollusks, particularly mussels such as *Perna perna*, are susceptible to MP ingestion primarily because of their filter-feeding behavior (Staichak et al., 2021). By processing large volumes of water to capture suspended particles, these organisms may retain MPs within edible tissues, which makes them especially relevant for evaluating seafood contamination and potential dietary exposure in coastal populations (Bom & Sá, 2022). In addition to particulate contamination by MPs, bivalves may also be exposed to plastic-related chemical contaminants such as bisphenol A (BPA). BPA can occur in the aquatic environment in dissolved form and may be taken up directly from the surrounding water, meaning that its presence in mussel tissues cannot be attributed exclusively to ingested microplastics (Chen et al., 2016). Because BPA is not covalently bound to the plastic matrix, it can leach from plastic materials into the surrounding environment, where it may become available for uptake by aquatic organisms (Bogdanović et al., 2025). As an Emerging Organic Contaminant (EOC), BPA is known for its endocrine-disrupting properties (Paolella et al., 2024), which can interfere with the metabolic and reproductive systems of marine biota even at environmentally relevant concentrations (Akhbarizadeh et al., 2021; Castellani et al., 2025; Lambré et al., 2023).

The risk to human health through the consumption of contaminated seafood is a major concern, particularly as regulatory agencies have significantly lowered safety thresholds for BPA exposure (Lambré et al., 2023). In 2015, the European Food Safety Authority (EFSA) reduced the tolerable daily intake (TDI) of BPA from 50 µg kg⁻^1^ bw day⁻^1^ to a temporary TDI (t-TDI) of 4 µg kg⁻^1^ bw day⁻^1^ (“Scientific Opinion on the risks to public health related to the presence of bisphenol A (BPA) in foodstuffs” 2015). More recently, in 2023, following a comprehensive re-evaluation of BPA toxicity, particularly its effects on the immune system, EFSA further reduced the TDI to 0.2 ng kg⁻^1^ bw day⁻^1^, a level 20,000 times lower than the 2015 limit (Lambré et al., 2023). This drastic reduction underscores the potential public health risks associated with dietary exposure to BPA, even at very low concentrations, and highlights the urgent need for updated risk assessments in commercially important seafood species.

Therefore, this study aimed to evaluate the occurrence of microplastics (MPs), representing particulate contamination, and bisphenol A (BPA), representing a plastic-related emerging chemical contaminant, in the tissues of the mussel *Perna perna* from southeastern Brazil. By assessing these two contaminant classes together, the study sought to provide complementary information on particulate and chemical contamination in edible mussels. In addition, human dietary exposure was estimated under different consumption scenarios to better understand the potential implications of these contaminants for food safety. This integrated approach provides occurrence and dietary exposure data for plastic-related contaminants in edible bivalves from a tropical South Atlantic region, where monitoring data for emerging contaminants in seafood remain limited.

## Materials and methods

### Study area and sample collection

**Fig. 1:**
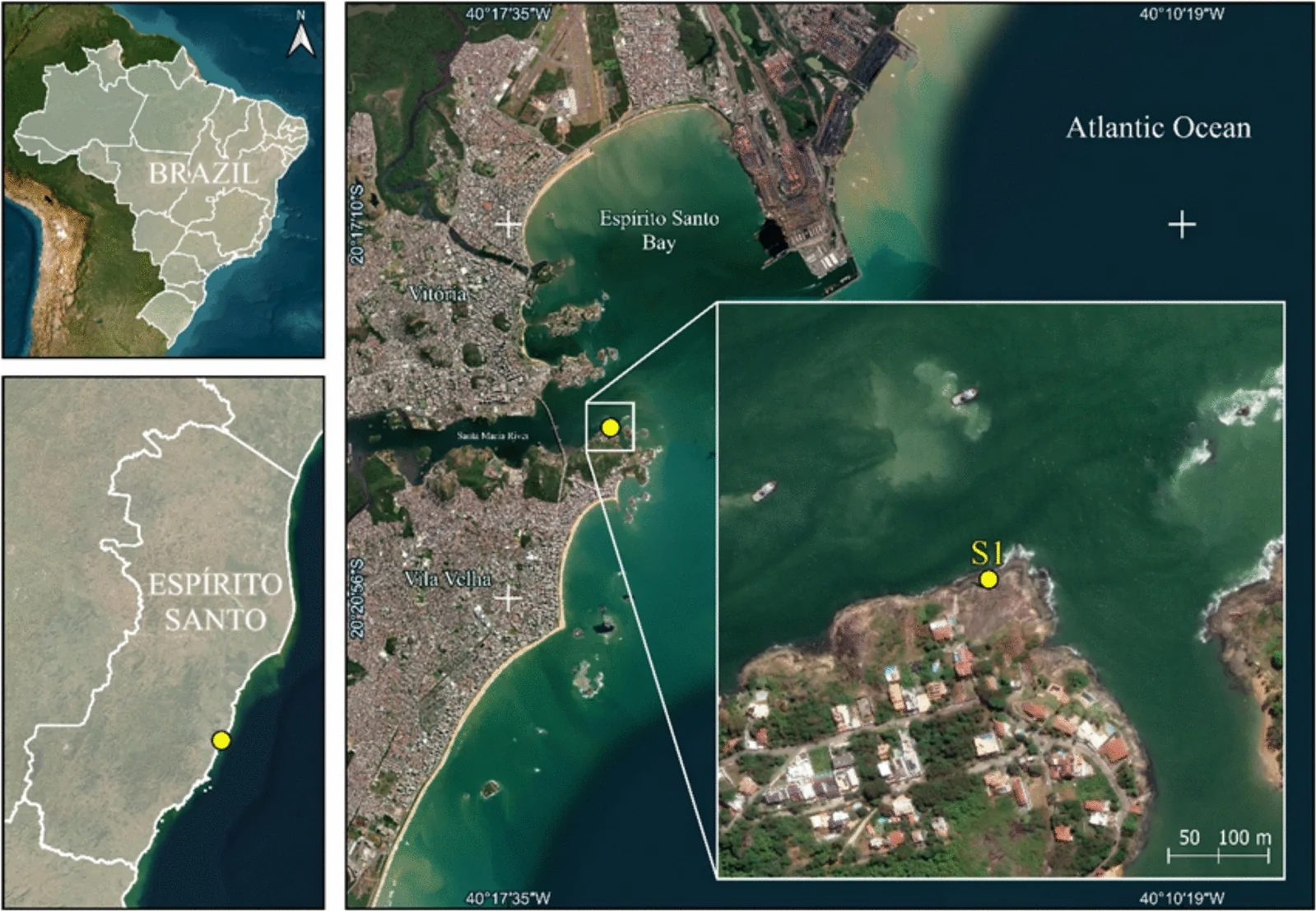
Location of the sampling site for *Perna perna* collection on the coast of Espírito Santo, southeastern Brazil. The map shows the regional location of Espírito Santo State within Brazil and a detailed view of Espírito Santo Bay, in the municipality of Vila Velha. The yellow marker (S1) indicates the exact sampling point where mussels were collected (20°19′13.1″S, 40°16′29.6″W)

Adult specimens of the brown mussel *Perna perna* (Linnaeus, 1758) were manually collected from natural banks in the intertidal zone of rocky substrates along the coast of Espírito Santo State, southeastern Brazil, specifically in Espírito Santo Bay, in the municipality of Vila Velha (20°19′13.1″S, 40°16′29.6″W) (Fig. 1). The species was selected because of its filter-feeding behavior and ability to accumulate contaminants from the surrounding environment, characteristics that support its use as a bioindicator of coastal contamination. In addition, *P. perna* is an edible bivalve of ecological and socio-economic relevance along the Brazilian coast. A single sampling site was selected because the study was designed as a site-specific assessment of contaminant occurrence and dietary exposure rather than as a spatial comparison among coastal areas. Therefore, the sampling design was intended to characterize contamination at this specific location and sampling period.

Vila Velha is a highly urbanized coastal municipality within the Greater Vitória metropolitan region, with approximately 468,000 inhabitants according to the 2022 Brazilian census. The municipality is part of a coastal-estuarine system influenced by intense urban occupation, port and navigation activities, and domestic and industrial inputs. The Vitória–Espírito Santo Bay system includes areas associated with the Port of Vitória and is subject to multiple anthropogenic pressures related to urbanization and maritime activities. Mussel harvesting and cultivation are also socio-economically relevant activities along the coast of Espírito Santo, where *P. perna* occurs naturally and is used for human consumption. These characteristics make the region environmentally relevant for assessing the occurrence of emerging contaminants in edible coastal organisms.

Sampling was conducted in October 2022, during the austral spring. A total of 60 individuals were collected, exhibiting an average shell length of 4.6 ± 0.438 cm and an average wet weight of 8.2 ± 2.1 g. Only individuals above the reported size at first sexual maturity for *Perna perna* in Vila Velha were selected. De Souza et al., (2019) reported sizes at first sexual maturity (L50) of approximately 25.2 mm for males and 25.1 mm for females in this region. Following collection, mussels were immediately placed in thermal boxes containing ice and transported to the Laboratory of Environmental Geochemistry (LabGam), Federal University of Espírito Santo (UFES), for subsequent processing.

### Sample preparation

Mussels were thawed and subjected to morphometric analysis, including shell length (SL, mm) and total weight (TW, g) (Abidli et al., 2021). Prior to dissection, shells were thoroughly cleaned with a soft-bristled brush and distilled water previously vacuum-filtered through 1.2-µm glass-fiber filters to remove epibionts and byssal threads, thereby minimizing external contamination. Each bivalve was carefully dissected using a clean stainless-steel scalpel to separate the soft tissues from the shells, and the soft tissue weight (STW, g) was recorded after draining excess intervalve water. The 60 individuals were divided into two groups: 30 specimens were allocated for BPA analysis and 30 for MPs identification, characterization, and quantification. For BPA, valid quantitative results were obtained for 30 samples with sufficient material/extract for analysis; samples without valid quantitative results were not treated as nondetects. MPs and BPA were measured in different subsets of individuals. Therefore, the study documents the occurrence of both contaminants within the sampled mussel population but does not allow direct individual-level associations between MP abundance and BPA concentration.

### Microplastic analysis

#### Extraction of microplastics

The extraction protocol was based on the alkaline digestion method described by Patterson et al. (2019). Soft tissues were rinsed with filtered distilled water to remove external particles. For tissue digestion, samples were placed in 1 L glass beakers, and 200 mL of 10% Potassium Hydroxide (KOH) was added (approximately three times the tissue volume). The beakers were covered with aluminum foil and incubated at 50 °C for 72 h, and complete digestion was verified visually by the absence of visible residual soft tissue in the digest.

Following digestion, a saturated saline solution (NaCl, 140 g L⁻^1^) was added (~700 mL) to promote the flotation of MP particles through density separation (Li et al., 2018). After 24 h of flotation at room temperature, the supernatant was filtered using a vacuum pump through glass fiber membrane filters (pore size: 1.2 μm). Filters were then placed in glass Petri dishes, covered, and dried at room temperature for subsequent analysis.

#### Identification and characterization

The MPs were classified according to size, color, and type following established criteria for plastic debris classification (Lusher et al., 2020; Thompson et al., 2009). Fibers were defined as individual elongated particles with a relatively uniform thickness, whereas tangled fibers were defined as aggregates composed of two or more intertwined or entangled filamentous particles forming a visually distinguishable bundle. Fragments were defined as irregular, nonfibrous particles without an elongated filamentous shape. Visual examination was performed using a Leica S8APO stereomicroscope equipped with a Leica MC170 HD. Particles were considered potential MPs based on morphological characteristics such as coloration, relatively uniform thickness, absence of visible cellular or organic structures, and morphology consistent with synthetic fibers or fragments. Fibers showing tapered ends or other morphological characteristics indicative of natural origin were excluded from the analysis. Suspected MPs were quantified and photographed, and images were processed using Leica Application Suite software, version 4.9.

Subset of 40 suspected MPs (21.74% of the 184 visually identified particles) was randomly selected for polymer identification by Raman microspectroscopy using a WITec Raman-AFM alpha300 microscope combination (Oxford Instruments-WITec, Ulm, Germany). Raman data acquisition was performed using the integrated Control FIVE 5.2 software and based on the Project FIVE 5.2 software package (WITec software, Germany). Raman spectra acquisition was conducted with a 532 nm laser, a 20 × visible microscope objective (Zeiss EC Epiplan 20 ×/0.4), a 200 µm confocal pinhole and a G1:600 grooves/mm BLZ = 500 nm grating with a spectral resolution of 1 cm^−1^. The sample was located using the optical microscope, focused, and analyzed by acquiring point spectra. The data were processed using baseline correction to identify the sample composition. The acquisition time was 1.0 s with 15 accumulations in air under ambient conditions (T = 293 K).

Raman spectroscopy has been widely applied for the identification and characterization of MPs due to its nondestructive nature, high sensitivity, and ability to provide detailed information on polymer composition (Araujo et al., 2018; Käppler et al., 2016; Lenz et al., 2015). Its high spatial resolution also enables the analysis of very small particles, including those that may be difficult to detect using techniques such as infrared spectroscopy (Käppler et al., 2016). Consequently, Raman spectroscopy has become an important analytical approach in studies investigating MP contamination in different environmental matrices (Araujo et al., 2018; Käppler et al., 2016; Lenz et al., 2015; Qin et al., 2024; Schymanski et al., 2018; Wu et al., 2024).

#### Quality assurance and control (QA/QC)

Measures were implemented to minimize background contamination throughout the analytical procedure. Procedural blanks containing no tissue were processed alongside each batch of samples using the same reagents and analytical steps applied to the samples. No suspected microplastics were detected in the procedural blanks; therefore, no blank correction was applied to the sample results. All glassware was rinsed with filtered distilled water and 70% ethanol, and the use of plastic equipment was avoided. All solutions were filtered through 1.2-μm filters prior to use. Analysts wore 100% cotton laboratory coats and latex gloves throughout the procedure, and samples remained covered with aluminum foil except during active manipulation and analysis. Recovery tests using reference microplastic particles were not performed; therefore, extraction efficiency was not experimentally quantified and this was considered a methodological limitation of the study.

### Bisphenol analyses

#### Sample pretreatment

Soft tissue samples allocated for chemical analysis were lyophilized for 96 h to remove moisture and concentrate the analytes. Wet mass before lyophilization and dry mass after lyophilization were recorded to allow conversion of BPA concentrations from dry weight to wet weight. After freeze-drying, the samples were homogenized, and an aliquot of approximately 200 mg dry weight (dw) was used for BPA extraction. Material was collected from the central portion of the tissue to minimize potential external contamination (Baralla et al., 2021; Vighi et al., 2023).

#### Bisphenol A extraction and instrumental analysis

For BPA determination, an aliquot of 200 mg of dried mussel powder (*n* = 30, which 18 were analyzed in duplicate) was extracted with 3 mL of methanol in an ultrasonic bath for 5 min. The extraction time was based on a previously reported BPA extraction procedure using methanol and ultrasonic treatment (Santos et al., 2023). Under the analytical conditions applied in the present study, the efficiency of the extraction procedure for BPA was further supported by matrix-spike recoveries ranging from 91.0 to 92.5%. The extracts were subsequently filtered through quantitative filter paper (8 µm porosity, JP 42 blue stripe, J. Prolab, São José dos Pinhais, PR, Brazil) into 4 mL glass vials, and the final volume was adjusted to 4 mL with methanol. The extracts were stored at 4 °C until instrumental analysis.

BPA determination was carried out using a GC-MS system (QP2010 Ultra, Shimadzu Corporation) equipped with a DB-5 ms capillary column (5% phenyl-95% methylpolysiloxane, 30 m × 250 μm × 0.25 μm; J&W Agilent). A 1 μL aliquot of each extract was injected in split mode (10:1), with the injector temperature set at 290 °C and purge flow at 3 mL min⁻^1^. The oven temperature program started at 150 °C with a 2 min hold, followed by a ramp of 30 °C min⁻^1^ up to 300 °C, with a final hold of 3 min. Helium (99.9999%) was used as the carrier gas at a constant flow rate of 1.2 mL min⁻^1^. The mass spectrometer operated in electron ionization mode (EI, 70 eV) under selected ion monitoring (SIM). The ion at *m*/*z* 213 was used for BPA quantification, whereas the ion at *m*/*z* 228 was used as a confirmation ion. The ion source and interface temperatures were maintained at 230 and 250 °C, respectively. The total run time was 10 min, with a solvent cut time of 2 min.

BPA identification was based on the agreement between sample and standard retention times, as well as on the monitored diagnostic ions under the same chromatographic conditions. Quantification was performed by external calibration using analytical BPA standard solutions at 0.005, 0.01, 0.02, 0.05, 0.10, and 0.15 µg mL⁻^1^. The analytical standard used was BPA (Sigma-Aldrich, product no. 239658; purity > 99%, HPLC). The calibration equation was *y* = 2,000,000*x*–14,468, with a coefficient of determination (*R*^2^) of 0.994 and a retention time of 6.775 min. Peak integration was performed using LabSolutions software (GCMS Solution version 4.20, Shimadzu Corporation). BPA concentrations were first calculated on a dry-weight basis and then converted to wet weight using the dry-to-wet mass ratio of each sample according to:

$${C}_{ww}={C}_{dw}\times \frac{{m}_{dw}}{{m}_{ww}}$$

where *C*_*ww*_ is the concentration in wet weight, *C*_*dw*_ is the concentration in dry weight, *m*_*dw*_ is the dry mass after lyophilization, and mww is the wet mass before lyophilization. Final BPA concentrations were expressed as μg kg⁻^1^ wet weight.

#### Quality assurance and quality control for BPA analysis

Measures were adopted throughout the analytical workflow to minimize external contamination during BPA determination, including the use of cleaned glass materials, gloves, and careful sample handling. Procedural blanks were analyzed concomitantly with the samples, and no BPA was detected above the method detection limit. Method performance was instead evaluated through matrix-spike recovery experiments. Matrix effects were evaluated through spiked experiments by adding 250 μL of BPA standard solution (0,1 μg mL^–1^) to the samples prior to BPA extraction. Recovery values ranged from 91.0 to 92.5%. The limit of detection (LOD) and limit of quantification (LOQ) were 0.0025 and 0.0075 μg mL^–1^, respectively. LOD is the lowest value of the analyte can be detected in relation to background noise. In general, LOD is defined by a signal-to-noise (S/N) = 3:1, and LOQ can be estimated as 3.0 times the LOD. The samples analyzed in duplicate presented standard deviation (SD) of ± 0.0001 to 0.0023, and the matrix spike recovery experiments had SD of ± 0.0175. For BPA method expressed in μg kg⁻^1^ dry weight, the tissue LOD and LOQ values are 50 and 150 μg kg⁻^1^ dw, respectively.

### Statistical analysis

Statistical analyses were performed using GraphPad Prism 8.0. Data normality was assessed using the Shapiro–Wilk test. Descriptive statistics were calculated for MP abundance and BPA concentrations, including mean, standard deviation, median, interquartile range, minimum and maximum values, as appropriate. For BPA, statistics were calculated using the 30 valid quantified samples after dry-to-wet weight conversion. Differences among MP morphotypes were evaluated using the Kruskal–Wallis test followed by Dunn’s post hoc test. Statistical significance was set at *p* < 0.05.

### Human dietary exposure assessment to microplastics

#### Estimated daily intake (EDI) calculation

Human dietary exposure to MPs through mussel consumption was estimated using the Estimated Daily Intake (EDI) approach under two consumption scenarios representing average and high shellfish intake (Ferrante et al., 2022; Zhou et al., 2023). The EDI was calculated according to the following equation:

$$\mathrm{E}\mathrm{D}\mathrm{I}=\frac{C \times \mathrm{I}\mathrm{R}}{\mathrm{B}\mathrm{W}}$$

where EDI is the estimated daily intake (particles kg⁻^1^ bw day⁻^1^), *C* is the MPs concentration in mussel tissue (MPs g⁻^1^ ww), IR is the ingestion rate (g day⁻^1^), and BW is the body weight of the consumer. A default adult body weight of 70 kg was adopted for the exposure assessment, following the recommendation of the EFSA Scientific Committee for adult populations when specific body-weight data are unavailable (EFSA Scientific Committee, 2012).

#### Mussel consumption scenarios

##### Scenario 1: Average national consumption

This scenario represents the average national consumption of mussels in Brazil, estimated at 50 g year⁻^1^, equivalent to 0.137 g day⁻^1^. This value reflects the low national per capita average for bivalves, considering the dilution of total production across the Brazilian population (Costa et al., 2021). Although overall fish consumption in Brazil has increased, the specific consumption of mussels remains relatively low and irregular on a national average (IBGE, 2025).

##### Scenario 2: High-consumption condition

This scenario simulates a high-consumption condition corresponding to a weekly portion of 200 g, equivalent to 28.57 g day⁻^1^. This value is representative of frequent consumers in coastal regions, where mussels are part of local dietary practices and gastronomy (Bom & Sá, 2022). A 200 g portion is consistent with typical seafood servings used in dietary exposure assessments.

#### Microplastic concentration in *Perna perna*

The MPs concentrations used in the calculations were derived from the analysis of *Perna perna* specimens collected in the present study. The mean MPs concentration (1.44 MPs g⁻^1^ ww) was applied for the average consumption scenario, while the 95th percentile concentration (3.84 MPs g⁻^1^ ww) was used for the high-consumption scenario, to represent a condition of higher exposure.

These values are consistent with recent scientific literature for bivalves on the Brazilian coast, where previous studies reported mean MP concentrations ranging from 1.64 MPs g⁻^1^ to 3.66 MPs g⁻^1^ in various bivalve species marketed in Brazil (Bom & Sá, 2022).

### Human dietary exposure and risk characterization for BPA

Human dietary exposure to BPA through *Perna perna* consumption was estimated using the same consumption scenarios and adult body weight adopted for the microplastic exposure assessment. The Estimated Daily Intake (EDI) was calculated according to:

$$\mathrm{E}\mathrm{D}\mathrm{I}=\frac{C \times \mathrm{I}\mathrm{R}}{\mathrm{B}\mathrm{W}}$$

where EDI is the estimated daily intake of BPA (ng kg⁻^1^ bw day⁻^1^), *C* is the BPA concentration in mussel tissue expressed on a wet-weight basis (ng g⁻^1^ ww), IR is the mussel ingestion rate (g day⁻^1^), and BW is the consumer body weight (70 kg).

Potential health concern was characterized by comparing the estimated BPA intake with the tolerable daily intake (TDI) of 0.2 ng kg⁻^1^ bw day⁻^1^ established by the European Food Safety Authority (EFSA) (Lambré et al., 2023). The hazard quotient (HQ) was calculated as:

$$\mathrm{H}\mathrm{Q}= \frac{\mathrm{E}\mathrm{D}\mathrm{I}}{\mathrm{T}\mathrm{D}\mathrm{I}}$$

An HQ < 1 indicates an estimated exposure below the reference value, whereas an HQ > 1 indicates that the reference value is exceeded under the assumptions of the evaluated consumption scenario.

## Results

### Microplastic abundance in *Perna perna*

**Fig. 2:**
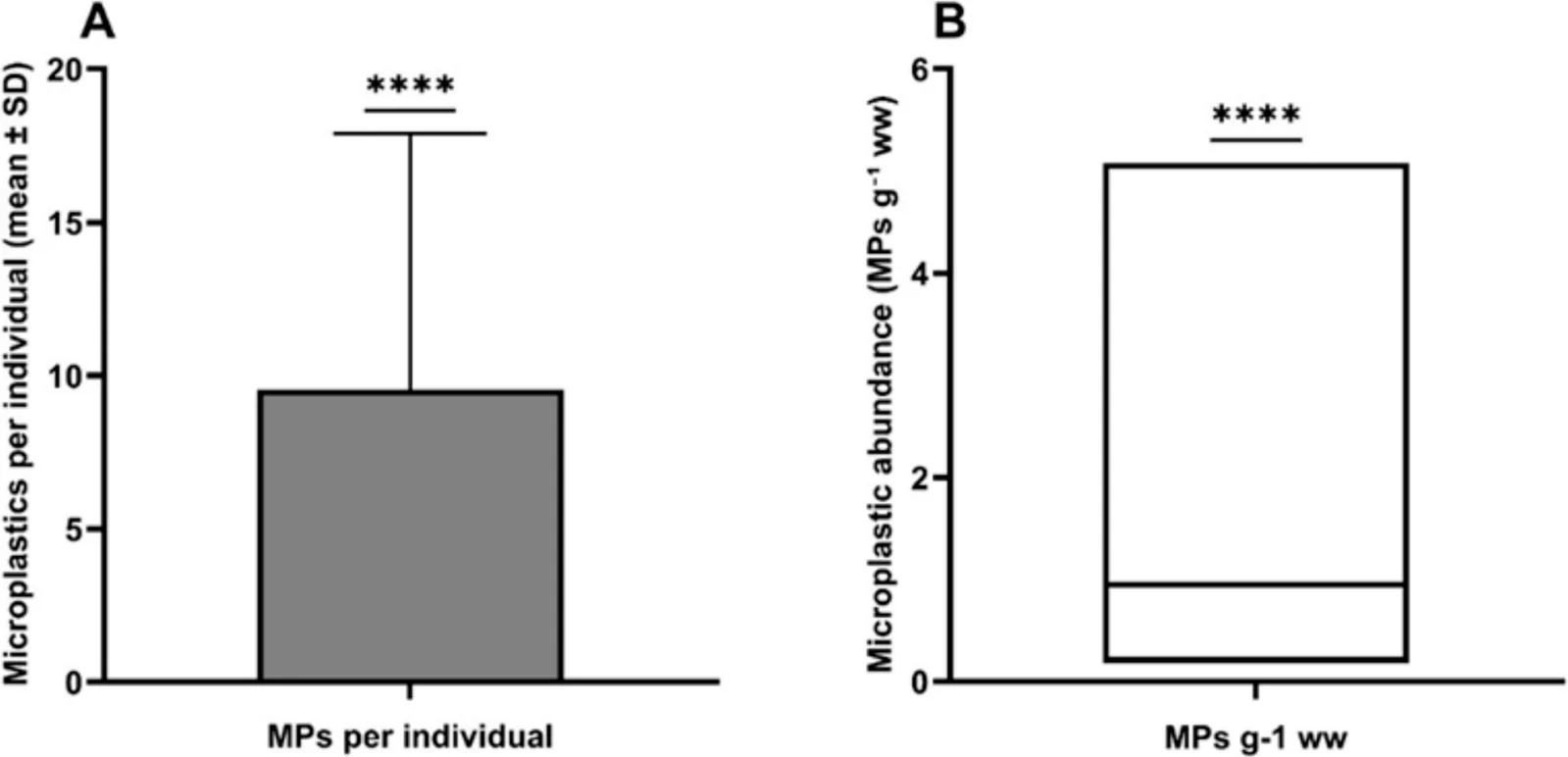
Microplastic abundance in the edible tissues of *Perna perna* (Linnaeus, 1758). **A** Mean abundance of microplastic particles per individual (MPs ind⁻^1^), with error bars representing the standard deviation (± SD). **B** Distribution of microplastic abundance expressed as particles per gram of wet weight (MPs g⁻^1^ ww). In panel (**B**), the box represents the interquartile range (25–75%); the central line indicates the median, and whiskers represent the minimum and maximum values (*n* = 30)

The edible soft tissues of *Perna perna* contained a mean MPs abundance of 6.1 ± 3.8 MPs ind⁻^1^ (Fig. 2A), corresponding to 1.44 ± 1.19 MPs g⁻^1^ ww (Fig. 2B) (*n* = 30). Particle counts varied considerably among individuals, indicating heterogeneous contamination levels within the sampled population. MPs concentrations ranged from 0.18 to 5.08 MPs g⁻^1^ ww, with a median of 0.95 MPs g⁻^1^ ww.

### Distribution of microplastic morphotypes

**Fig. 3:**
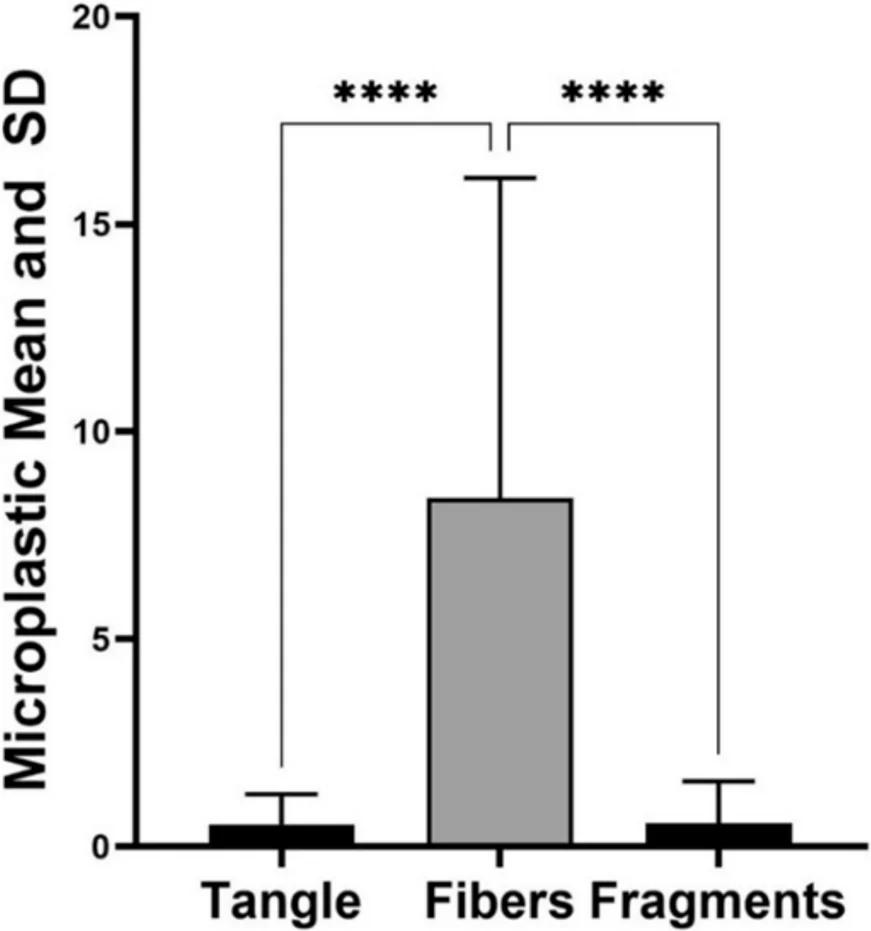
Relative frequency (%) of microplastic morphotypes identified in the tissues of *Perna perna* (Linnaeus, 1758), including tangled fibers, fibers, and fragments. Significant differences between groups were determined using the Kruskal–Wallis test followed by Dunn’s multiple comparison test (*p* < 0.05)

A total of 285 suspected MP particles were identified in the edible tissues of *Perna perna*, comprising three main morphotypes: tangled fibers, fibers, and fragments. Fibers were the dominant morphotype, accounting for 252 particles (88.42%), followed by fragments with 17 particles (5.96%) and tangled fibers with 16 particles (5.61%) (Fig. 3). Statistical analysis revealed significant differences among morphotypes (Kruskal–Wallis test, *p* < 0.05). Dunn’s post hoc test indicated that fibers were significantly more abundant than tangled fibers and fragments.

### Color distribution

**Fig. 4:**
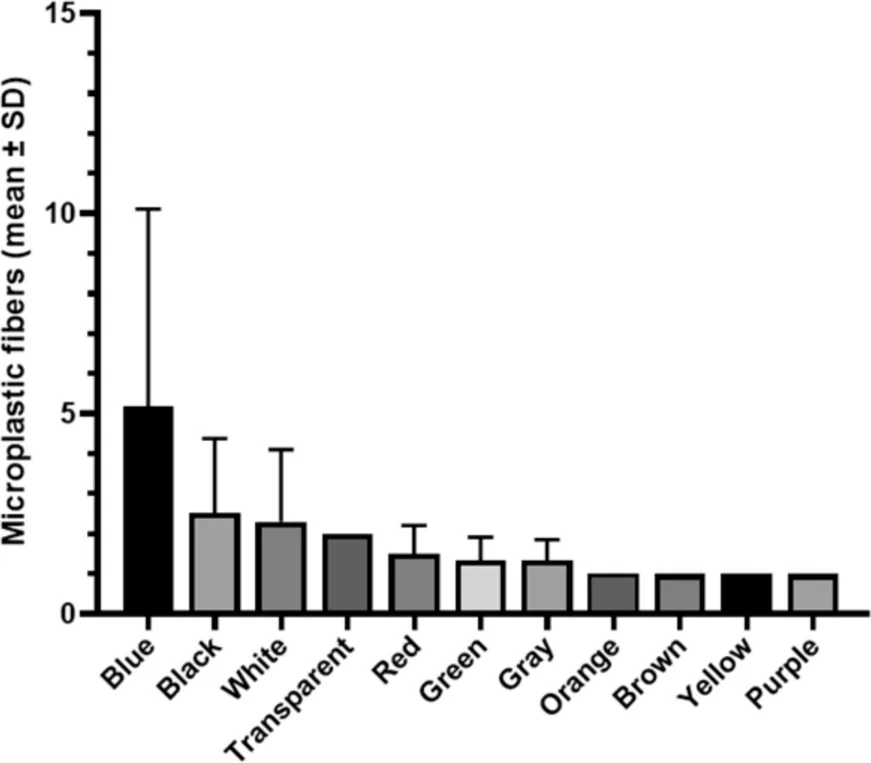
Color distribution of filament-type microplastics identified in the tissues of *Perna perna* (Linnaeus, 1758). Bars represent the mean abundance (± standard deviation) of fibers according to color categories. Blue and black fibers were the most frequent color classes observed in the analyzed samples

The color distribution of suspected MPs showed considerable variation among samples. Blue particles were the most abundant, accounting for 145 particles (51.06%), followed by black particles with 58 particles (20.42%) and white particles with 53 particles (18.66%). Gray particles accounted for 8 particles (2.82%), whereas green, yellow, and purple particles each accounted for 4 particles (1.41%). Red particles accounted for 3 particles (1.06%), transparent and brown particles each accounted for 2 particles (0.70%), and orange particles were the least frequent, with 1 particle (0.35%) (Fig. 4).

**Fig. 5:**
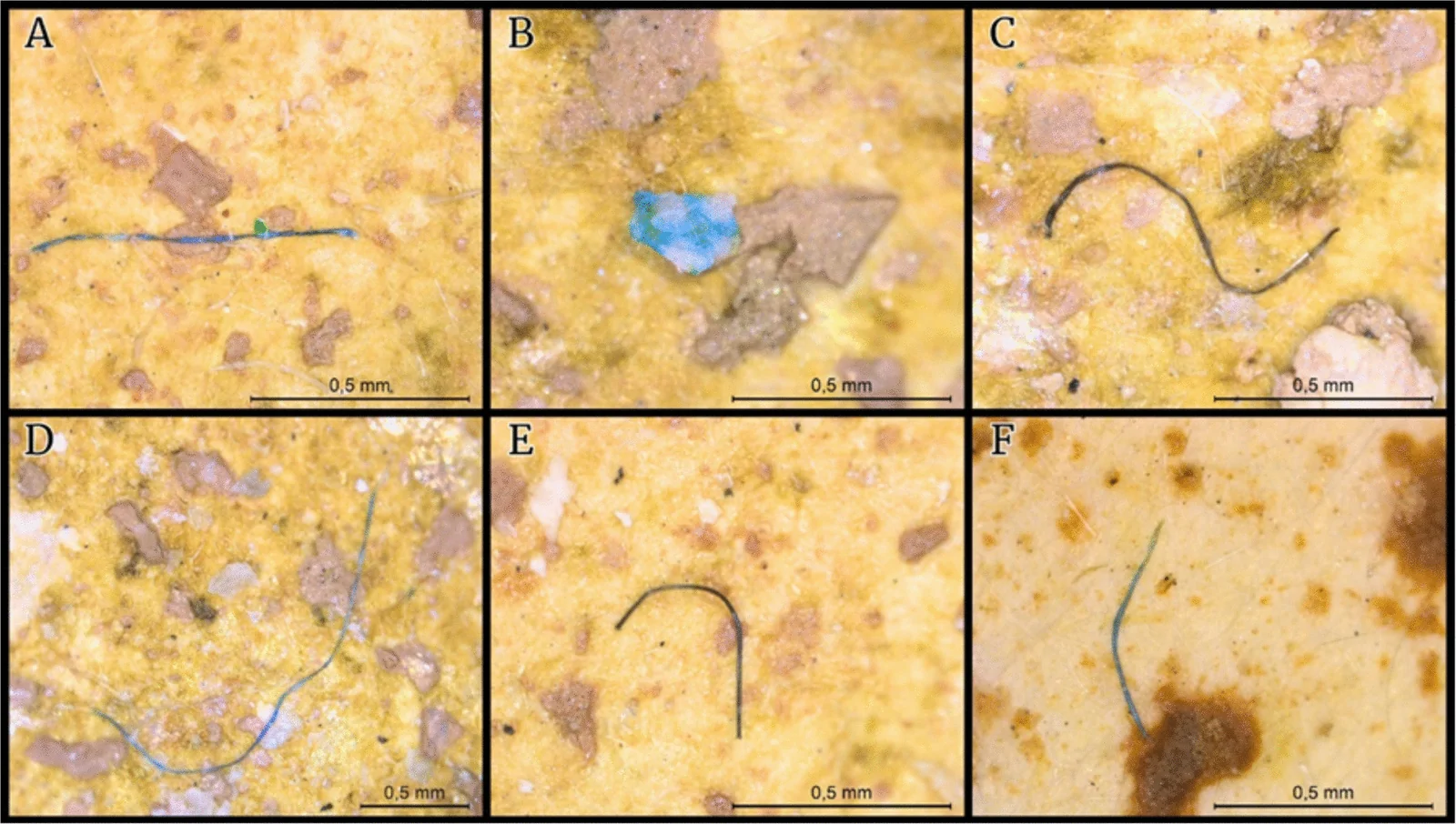
Representative stereomicroscopic images of microplastics isolated from *Perna perna* tissues, showing different morphotypes and colors, predominantly fibers. Panels illustrate examples of particles identified in the samples: **A**, **D**, **F** blue fibers; **B** blue fragment; **C**, **E** black fibers. Scale bars = 0.5 mm

Representative stereomicroscopic images of the MP particles isolated from *Perna perna* tissues are presented in Fig. 5, illustrating the main morphotypes and colors identified in the samples, predominantly blue and black fibers.

### Microplastic characterization

**Fig. 6:**
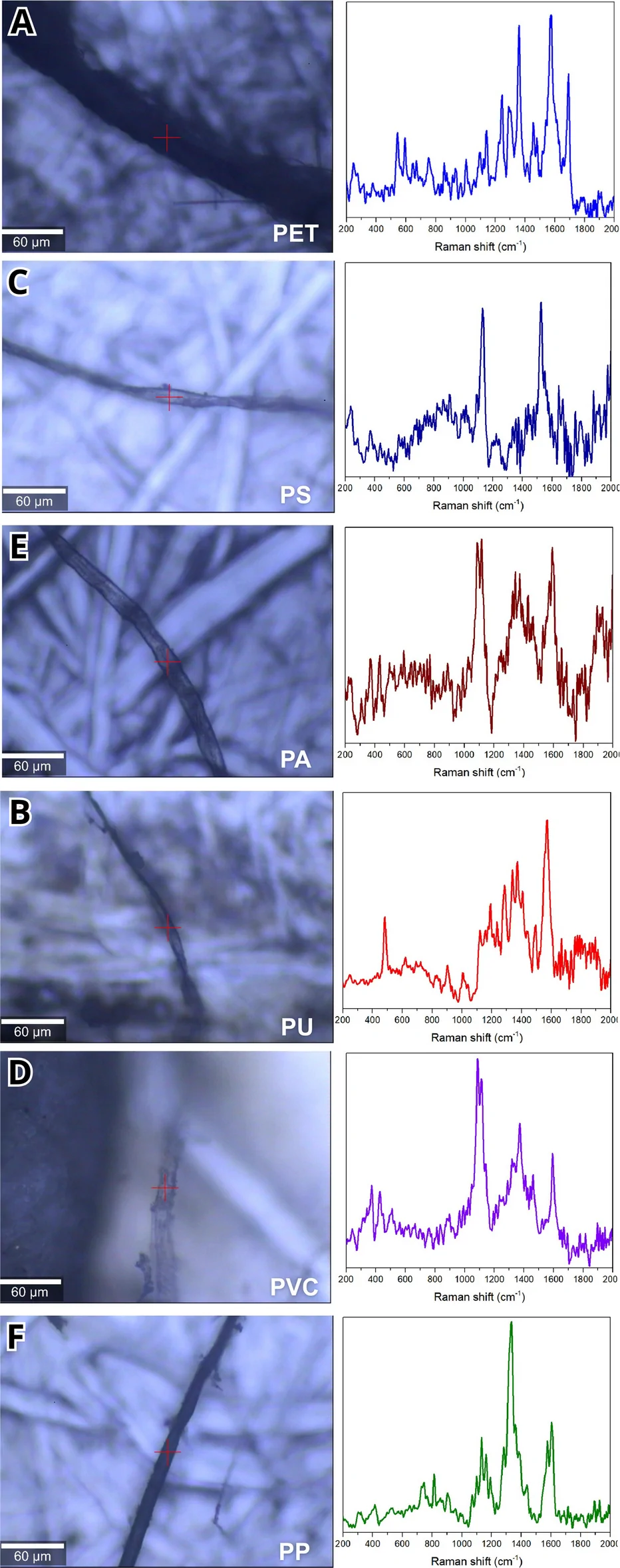
Representative Raman spectra and corresponding images of polymer particles identified in *Perna perna* tissues: **A** polyethylene terephthalate (PET), **B** polyurethane (PU), **C** polystyrene (PS), **D** polyvinyl chloride (PVC), **E** polyamide/nylon (PA), and **F** polypropylene (PP). Raman spectra are shown over the range of 200–2000 cm⁻^1^

Raman spectroscopy identified six polymer types among the 40 analyzed particles. Polyethylene terephthalate (PET) was the most frequent polymer, accounting for 17 particles (42.5%), followed by polyurethane (PU), with 10 particles (25%). Polyvinyl chloride (PVC), polystyrene (PS), and polypropylene (PP) each accounted for 4 particles (10%), whereas nylon (PA) was identified in 1 particle (2.5%) (Fig. 6) (Nesterovschi et al., 2023, 2025).

### Occurrence of BPA in *Perna perna*

**Fig. 7:**
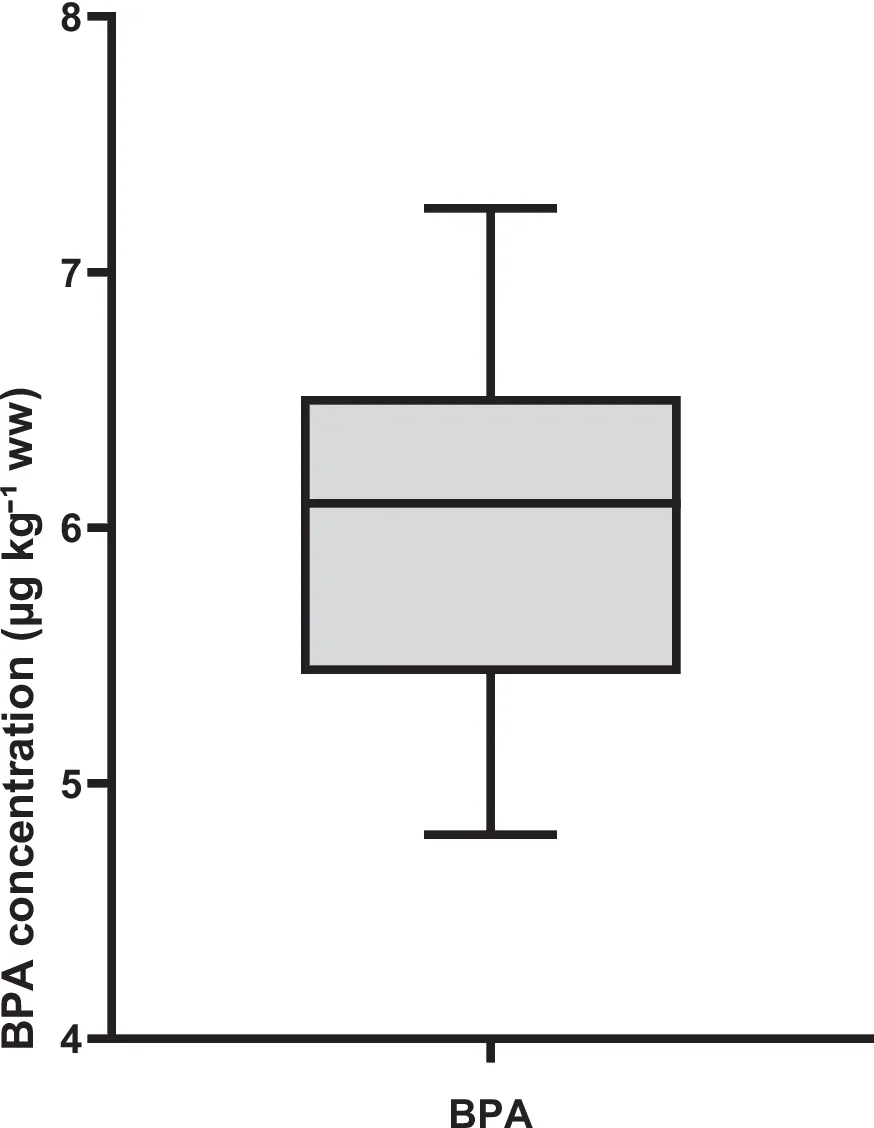
Distribution of bisphenol A (BPA) concentrations in *Perna perna* expressed on a wet-weight basis (μg kg⁻^1^ ww) after dry-to-wet weight conversion. The box represents the interquartile range (Q1–Q3), the horizontal line indicates the median, and whiskers represent the minimum and maximum values of the 18 valid quantified samples

BPA was quantified in 30 samples, which 18 were valid *Perna perna* samples analyzed in duplicate. The BPA values ranged from 146.69 to 367.48 μg kg⁻^1^ dw for all replicates of *Perna perna* samples (*n* = 48). On a dry-weight basis, BPA concentrations (*n* = 18, valid samples) ranged from 160.75 ± 5.89 to 203.34 ± 1.76 μg kg⁻^1^ dw, with a median of 180.57 μg kg⁻^1^ dw and a mean of 181.07 ± 12.95 μg kg⁻^1^ dw. All BPA values were higher than the LOD (50 μg kg⁻^1^ dw) and LOQ (150 μg kg⁻^1^ dw), with exception for one replicate 146.69 μg kg⁻^1^ dw. After conversion to wet weight using the sample-specific dry-to-wet mass ratios, concentrations ranged from 4.80 to 7.25 μg kg⁻^1^ ww, with a median of 6.10 μg kg⁻^1^ ww (Q1–Q3: 5.54–6.49 μg kg⁻^1^ ww) and a mean of 6.02 ± 0.64 μg kg⁻^1^ ww (Fig. 7).

### Human dietary exposure assessment of microplastics

**Fig. 8:**
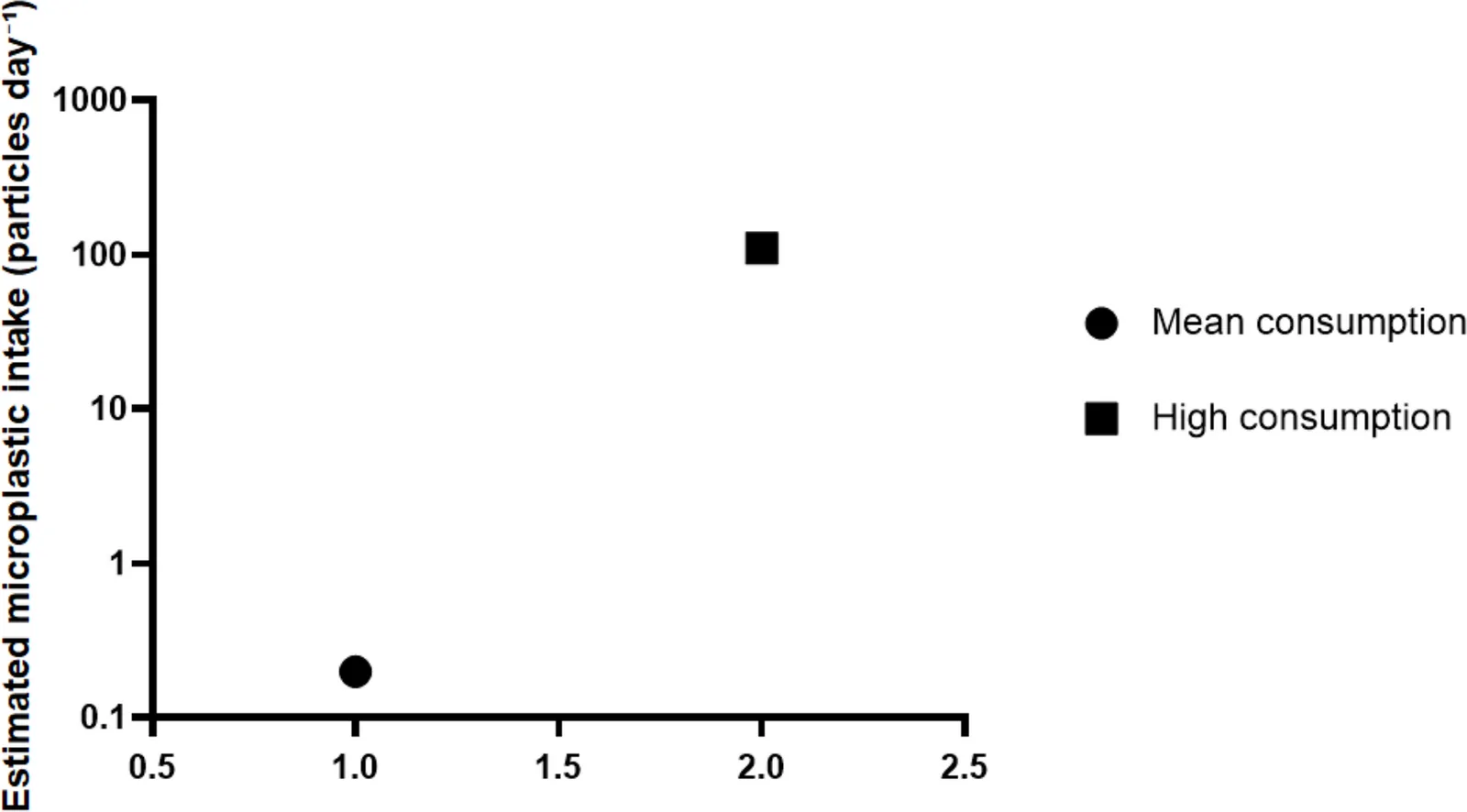
Estimated human exposure to microplastics through mussel (*Perna perna)* consumption in Brazil. The graph illustrates the estimated daily intake (EDI) of MPs under two consumption scenarios: (i) average national consumption (50 g year⁻^1^, equivalent to 0.137 g day⁻^1^), and (ii) a high-consumption scenario, corresponding to a weekly portion of 200 g (28.57 g day⁻^1^). Calculations were based on MPs concentrations observed in *Perna perna* specimens analyzed in this study (mean of 1.44 MPs g⁻^1^ ww for the average scenario and 95th percentile of 3.84 MPs g⁻^1^ ww for the high-consumption scenario)

Human dietary exposure to MPs was estimated under the two consumption scenarios described in the “Human dietary exposure assessment to microplastics” section. Based on the mean MP concentration observed in *Perna perna* (1.44 MPs g⁻^1^ ww), the estimated intake for the average-consumption scenario was approximately 0.20 particles day⁻^1^ (0.0028 particles kg⁻^1^ bw day⁻^1^). In contrast, considering the high-consumption scenario and the 95th percentile concentration (3.84 MPs g⁻^1^ ww), the estimated intake increased to 109.81 particles day⁻^1^ (1.57 particles kg⁻^1^ bw day⁻^1^) (Fig. 8).

### Human dietary exposure and risk characterization for BPA

Under the average-consumption scenario, the estimated daily intake of BPA was 0.0118 ng kg⁻^1^ bw day⁻^1^, corresponding to an HQ of 0.06. This value remained below the EFSA TDI of 0.2 ng kg⁻^1^ bw day⁻^1^ (Lambré et al., 2023), indicating low concern for average consumers under the assumptions used in this screening assessment.

Under the high-consumption scenario, the estimated daily intake of BPA was 2.46 ng kg⁻^1^ bw day⁻^1^, corresponding to an HQ of 12.30 and exceeding the EFSA TDI. However, this result should be interpreted cautiously because it represents a conservative screening scenario based on a high-consumption rate, the stringent EFSA reference value, and a relatively limited quantitative dataset (*n* = 30). Therefore, the HQ > 1 indicates exceedance of the adopted reference value under the assumptions of this scenario rather than evidence of an established health effect.

**Fig. 9:**
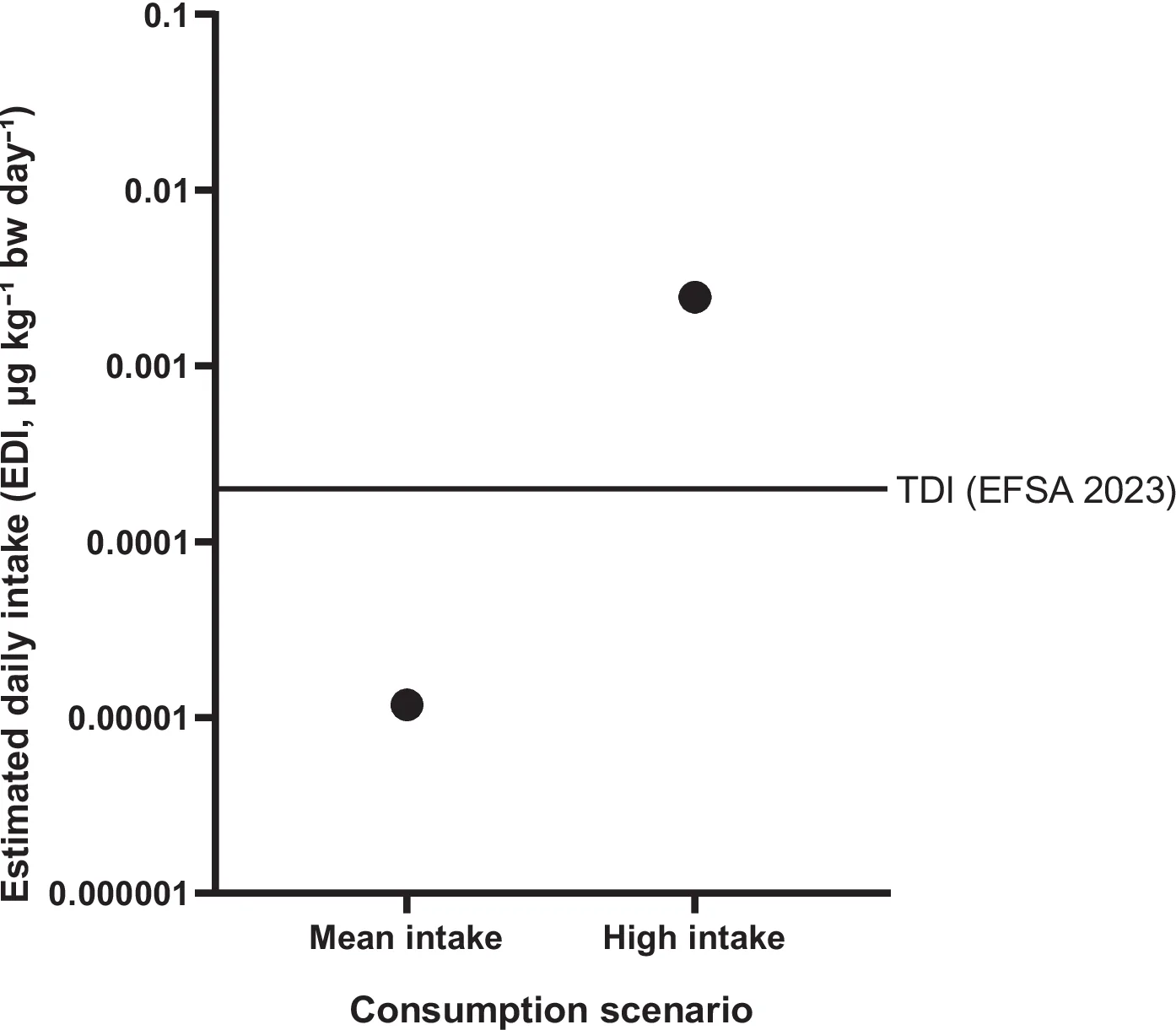
Estimated daily intake (EDI) of BPA through the consumption of *Perna perna* under average- and high-consumption scenarios. The horizontal line represents the tolerable daily intake (TDI = 0.2 ng kg⁻^1^ bw day⁻^1^) established by the European Food Safety Authority (EFSA, 2023). Values above the reference line indicate that the estimated exposure exceeds the adopted TDI under the assumptions of the evaluated consumption scenario

The comparison between estimated exposure and the EFSA toxicological reference value is illustrated in Fig. 9. The results show that BPA exposure remained below the TDI under average consumption but exceeded the reference value under the high-consumption scenario. These findings highlight the importance of considering consumption patterns when assessing dietary exposure to emerging contaminants such as BPA in seafood.

## Discussion

Edible *Perna perna* mussels collected at the investigated site in Espírito Santo Bay contained both microplastics (MPs) and BPA, indicating that coastal shellfish may represent a route of dietary exposure to plastic-related contaminants. These findings are relevant to dietary exposure assessment because mussels are generally consumed as whole soft tissues, and MPs detected in edible tissues may therefore contribute to human exposure. However, in the absence of established toxicological reference values for MPs, the present study does not allow a formal characterization of health risk associated with MP ingestion.

The MP abundance observed in the present study indicates contamination of edible *P. perna* at the sampled site in Espírito Santo Bay. Given the site-specific and single-period sampling design, these findings should be interpreted as a local snapshot of contamination rather than as representative of the entire bay or southeastern Brazilian coast. The predominance of fibers agrees with the pattern commonly reported for mussels and other seafood products intended for human consumption, in which filamentous MPs are usually the dominant morphotype (Bom & Sá, 2022; J. Li et al., 2016; Mercogliano et al., 2020; Van Cauwenberghe & Janssen, 2014). The predominance of blue and black fibers further suggests the contribution of urban and coastal anthropogenic sources, including synthetic textiles, ropes, fishing-related materials, and wastewater-derived microfibers (Costa et al., 2023; Otegui et al., 2024).

The predominance of fibers and the urbanized setting of the study area suggest the contribution of anthropogenic coastal sources, including wastewater, port-related activities, and fishing materials (Dalbó et al., 2025; Gatidou et al., 2019).

At the regional scale, the mean MP abundance observed in the present study (1.44 ± 1.19 MPs g⁻^1^ ww) falls within the range reported by Costa et al. (2023) for pooled soft tissues of four bivalve species collected from urban beaches in Vitória, southeastern Brazil (0.40–7.85 MPs g⁻^1^). In that study, MP abundance in individually analyzed bivalves ranged from 7.30 ± 1.11 to 31.22 ± 7.32 MPs ind⁻^1^, with a 100% frequency of occurrence. A subsequent study conducted at the same sites after a beach nourishment event reported a substantial increase in MP contamination, with 7097 particles recovered from bivalve tissues and a 171% increase in the total number of MPs compared with the previous sampling period (de Paula et al., 2025). In the pooled-tissue analysis, de Paula et al. (2025) reported a maximum concentration of approximately 15.22 MPs g⁻^1^ for *Mytella strigata*, while *P. perna* showed the highest total number of MPs among the individually analyzed species at sampling point S1 (834 particles in 10 individuals). Differences among these studies may reflect temporal variation, local anthropogenic pressures, coastal interventions such as beach nourishment, interspecific differences in particle uptake, and methodological factors. Therefore, although the MP abundance observed in the present study is within the range previously reported for bivalves from the region, direct quantitative comparisons should be interpreted cautiously because of differences in species, sampling design, and analytical approach.

The predominance of fibers is also biologically plausible given the feeding strategy of mussels, which process large volumes of water and efficiently retain suspended particles, including fibrous MPs (Khanjani et al., 2023; Staichak et al., 2021; Xu et al., 2024). Previous studies have shown that MPs may affect feeding, oxidative balance, immune responses, energy allocation, and tissue integrity in marine bivalves (Cole et al., 2020; Sendra et al., 2021). Although the present study did not assess biological effects in *P. perna*, the recurrent detection of MPs in edible tissues remains relevant because consumers may be exposed not only to the particles themselves but also to associated chemicals and additives. Plastic materials represent an important source of bisphenols in aquatic environments because these compounds are widely used in the manufacture of polycarbonate plastics, epoxy resins, and other polymer-based products. Because BPA is not covalently bound to the polymer matrix, it may leach from plastic materials into surrounding water and become available for uptake by aquatic organisms (Bogdanović et al., 2025; Paolella et al., 2024).

BPA was quantified in 30 samples, which 18 were valid *Perna perna* samples, confirming its occurrence in edible mussel tissues from Espírito Santo Bay. Samples without valid quantitative results were not considered nondetects because the limitation was related to insufficient material/extract rather than analytical nondetection. Therefore, the BPA dataset should be interpreted as a quantitative subset of the individuals initially allocated for chemical analysis.

After dry-to-wet weight conversion, BPA exposure was below the EFSA 2023 TDI for average mussel consumption (HQ = 0.06) but exceeded the reference value under the high-consumption scenario (HQ = 12.30). This finding indicates that frequent consumers may be more vulnerable to BPA exposure through contaminated mussels, reinforcing the importance of considering local consumption patterns in seafood risk assessment.

This result deserves attention because BPA is a well-established endocrine-disrupting compound and seafood can contribute to dietary exposure when contamination occurs in edible matrices (Lambré et al., 2023; Paolella et al., 2024). The occurrence of BPA and other bisphenols in mussels and other seafood products has been documented in recent studies from different coastal settings, including shellfish used for food safety and human health risk assessment (Akhbarizadeh et al., 2021; Baralla et al., 2021; Borriello et al., 2025; Castellani et al., 2025; Di Bella et al., 2025; Rios-Fuster et al., 2022).

Both MPs and BPA were detected within the sampled mussel population, although they were measured in different individuals. Therefore, the present dataset does not support direct individual-level associations between MP abundance and BPA concentrations, nor does it demonstrate that the BPA detected in mussel tissues originated from the MPs identified in the study. Instead, the results provide complementary evidence of particulate and chemical plastic-related contamination at the sampled site.

Although toxicological thresholds for dietary MP intake are still not established, the present exposure estimates indicate that MP intake through mussel consumption may vary substantially according to consumption frequency. This is consistent with recent studies emphasizing that contamination in edible aquatic tissues should be translated into consumer exposure metrics and interpreted in the context of realistic dietary habits (Jin et al., 2025; Mukhopadhyay & Valsalan, 2024).

From a food monitoring perspective, the present findings support continued surveillance of MPs and BPA in edible coastal shellfish, while recognizing that the occurrence of MPs indicates dietary exposure rather than a quantified toxicological risk. This is particularly relevant in regions where mussels are regularly consumed and where local contamination may disproportionately affect frequent consumers (Bom & Sá, 2022). Recent regulatory analyses have highlighted that contaminants of emerging concern remain incompletely covered by current seafood safety frameworks, despite increasing evidence of their occurrence and relevance to dietary exposure. De Witte et al. (2022) specifically noted that seafood may represent an important exposure route for contaminants for which food limits are absent or still under development, including plastic-related contaminants such as bisphenols. In this sense, BPA contamination in edible mussels should be viewed not only as a local coastal contamination issue, but also as part of a broader challenge in seafood surveillance, risk assessment, and regulatory updating.

This study has some limitations that should be acknowledged. First, MPs and BPA were measured in different subsets of mussels, preventing direct individual-level association analyses between both contaminants. Second, valid quantitative BPA results were obtained for 18 samples with sufficient material/extract, and samples without valid quantitative results were not interpreted as non-detects. Third, the exposure assessment is screening-based and depends on measured concentrations and predefined consumption scenarios rather than on a full probabilistic framework. Finally, the study was restricted to a single sampling site and a single sampling period. Therefore, the observed MP and BPA concentrations should be interpreted as a site- and time-specific snapshot rather than as representative of Espírito Santo Bay or the southeastern Brazilian coast. Broader spatial and temporal surveys are required to assess seasonal variability and determine the representativeness of the contamination patterns observed here.

Overall, the present findings indicate that edible *P. perna* from Espírito Santo Bay may represent a relevant route of dietary exposure to both MPs and BPA. These results support the need for continued surveillance of emerging contaminants in edible coastal shellfish, particularly in high-consumption settings.

## Conclusion

In conclusion, edible *Perna perna* mussels from southeastern Brazil contained both microplastics and BPA, indicating that shellfish may represent a relevant route of dietary exposure to plastic-related contaminants. After dry-to-wet weight conversion, the estimated BPA intake under average mussel consumption remained below the current EFSA reference value, whereas the high-consumption scenario exceeded this threshold, highlighting a potential concern for frequent consumers. These findings support the need for surveillance of emerging contaminants in edible coastal shellfish and for cautious food safety communication in high-consumption settings. Further studies should expand the geographical coverage of shellfish monitoring and strengthen analytical validation for BPA determination in edible marine organisms.

## Data Availability

The datasets generated during and/or analyzed during the current study are available from the corresponding author on reasonable request.
